# Optical Triangulation-Based Microtopographic Inspection of Surfaces

**DOI:** 10.3390/s120404399

**Published:** 2012-03-29

**Authors:** Manuel F. M. Costa

**Affiliations:** Centro de Física, Universidade do Minho, Campus de Gualtar, Braga 4710-057, Portugal; E-Mail: mfcosta@fisica.uminho.pt; Tel.: +351-253-604-070; Fax: +351-253-604-061

**Keywords:** microtopography, optical triangulation, non-invasive inspection, rugometry

## Abstract

The non-invasive inspection of surfaces is a major issue in a wide variety of industries and research laboratories. The vast and increasing range of surface types, tolerance requirements and measurement constraints demanded during the last decades represents a major research effort in the development of new methods, systems and metrological strategies. The discreet dimensional evaluation the rugometric characterization and the profilometric inspection seem to be insufficient in many instances. The full microtopographic inspection has became a common requirement. Among the different systems developed, optical methods have the most important role and among those triangulation-based ones have gained a major status thanks to their flexibility, reliability and robustness. In this communication we will provide a brief historical review on the development of optical triangulation application to the dimensional inspection of objects and surfaces and on the work done at the Microtopography Laboratory of the Physics Department of the University of Minho, Portugal, in the development of methods and systems of optical triangulation-based microtopographic inspection of surfaces.

## 1. Introduction

The non-invasive dimensional characterization of objects and surface is an issue of utmost importance in a variety of research works and in a wide range of industries. Not only are intrusive contact based systems no longer an acceptable option, but also the characterization of the relief structure of surfaces by the calculation of meaningful parameters of statistical nature like roughness (R_q_ R_a_, …), kurtosis, correlation length, zero crossings, …, is frequently not enough. More and more often, the integral reproduction or inspection of the three dimensional structure of surface's relief is needed. In industry this is especially true as competition and consumer's quality awareness grow.

Strict control of the production process, knowing, as well as possible, how any and every material or parts behave along the processing phases is crucial. Most of the surfaces involved are optically rough and in an extreme variety of shapes and types: from hard, stable, to soft ones with very little self consistency; with random height distributions or clearly non-isotropic; formed by just one component or by several areas made of different materials. Also different types of inspection tasks must be performed with high or lower resolution and or dynamic range requirements, but always reliably and in a fast and inexpensive way [1–3]. Furthermore the performance of the existing topographic inspection system is highly dependent on the inspection task and the kind of surface to be inspected. Usually the number of surface types that a particular system can successfully inspect is quite limited [3–5].

Optical triangulation in different approaches allow the establishment of metrological systems that by their inherent relative simplicity, robustness and reliability can cope with most modern requirements of the non-invasive inspection of objects and surfaces, both smooth or rough.

### 1.1. Brief Historical Revision of the State of the Art

The micro-inspection by optical, non-invasive, methods of the relief structure of surfaces and objects has nowadays a great, and surely increasing, importance. During the past decades new approaches to well-established methods like triangulation, both in area and discreet aproaches, were explored to meet the new requirements. Below we will provide a brief historical review on this subject focusing on optical triangulation comparing the main triangulation based dimensional inspection methods: moiré that combines interferometry and triangulation, contrived or structured lightning and discreet active triangulation.

Back in the 70s, the recent developments in electronics, lasers and optical and optoelectronic sensors allowed us to start to positively respond to the increasing requests from industry and investigation laboratories, of rugometric profilometric or even microtopographic (more recently) evaluation of surfaces on a non-destructive way. Optical methods were explored and/or developed and different systems were created and tested: based on the speckle effect [6–9]; Sprague [7] introduced the use of white light speckle on measuring roughness; scanning microscopy [10–12]; interferometry (from 1976 Bennett published an in depth analysis of the existing systems for the inspection of non-rough optic surfaces [13], including holographic interferometry [14] and Moiré's techniques [15,16] of increasingly facilitated development, inclusively on real time applications, by the emergence of increasingly efficient and accessible computer systems and software platforms); fringe projection including with the introduction by Indebetouw [17] of the concept of sliding fringes; or laser scanning by Smolka [18]; and an profilometry triangulation based system for measuring displacements, said in real time, by Sawatari [19] in 1976.

The 80s started with the impact of the recent emergence of confocal microscopy [18,20–22]. Various studies emerged about the use of the speckle phenomenon, but also on how to reduce that effect that limits the resolution of measures by laser based techniques, with Lim providing an important review of the theme in 1981 [18,23].

In 1984 Bertani published the use of photodetector arrays in locating luminous points (spots) as the ones of interest in active triangulation [24]; Rioux [25] presented a triangulation approach in which the sample scanning is synchronized with data acquisition, permitting one to maintain a good resolution with the use of reduced triangulation angles; and Tanwar [26] registered a setup in specular triangulation geometry at 45° allowing the measurement of displacements in the nanometer range. In 1986, Seitz [27] proposed the use of an optic triangulation system in 3-D coordinate measurement tables. In 1987, Durnin [28] published an interesting article about the generation of non-diffractable beams, the Bessel beams, of potential interest in active triangulation systems [29]. Hausler drew attention to the limitations in resolution the use of coherent light imposes in laser interferometers and profilometers [30,31].

In 1994, Dorsch and Hausler *et al.* discussed the fundamental uncertainty in measuring distances by laser triangulation [32] establishing that the minimum uncertainty will be defined by the speckle according to the principle of uncertainty of Heisenberg. The reference to tridimensional inspection of surfaces, and not only the 2D profilometric inspection as until then, was made independently by Costa, Molesini and Kimura [33–35].

In surface dimensional metrology, triangulation was not regarded as a state of the art technique but in general as a minor technique specially used on measuring distances or larger objects, even reaching several meters (range sensing), and with resolutions typically in the order of millimeters or at best several hundredths of a millimeter, therefore up to orders of magnitude higher than that usually required in the inspection of surfaces. In both domains in which the dimensional inspection of surfaces is more frequently and long time applied, in industry and even in research, that is in metal-mechanics and in optics, other methods and systems were preferred (in fact it was from that experience that most of the standards in dimensional metrology were drawn), namely stylus contact systems and interferometric systems. With the increasing requirements of non-contact non-invasive inspection researchers begin working on the study and development of the commonly called optical stylus, focus detection based systems, and not on triangulation. Only in the late 80s and early 90s the problems of the development of non-contact surface inspection optical systems based on triangulation was theoretically explored in depth and systems started to be developed and marketed. Starting in 1988, Bennet from the US Naval Air Warfare Center, published several review papers [36] about methods and systems of measurement and characterization of surfaces that are a reference to the people working on optical metrology. The first reference made to work on optical triangulation methods and systems was to a paper by Costa published in 1993 in *Applied Optics* [37]. In 1996, Costa [38] demonstrated that the calibration of optical triangulation systems can be made with the sample to be inspected, itself. In 1999, Zeng [39] proposed the use of triangulation in time-of-scan configurations and with two laser beams for measuring the position of moving objects. In 2000, Wang [40] proposed a system in which triangulation is combined with the analysis of scattered light by the surface, achieving resolutions, in roughness measurements, between 5 nm and 0.1 mm with height ranges up to 600 mm.

Since the beginning of the 21st century new applications have been explored. In 2001, Moore proposed the use of an active triangulation system for measurement of distances underwater in the calibration of a barometric laser scanning system [41]. In 2002, Chen [42] also published a paper studying the application of optic triangulation in measurement of distances underwater. Elazar presented, in 2002, a triangulation system using optic fibers for measurement of displacements in synchrotrons [43]. Lombardo [44] proposed, in 2003, the measurement of the scanning time on a triangulation system with beam scanning to measure displacements ranging from 1 to 20 cm, with resolutions on the order of tenths of a millimeter. In 2004, Liu [45] demonstrated a triangulation sensor using a diffraction network and two quadrant detectors. Although the basic principle of the measurement of distances by triangulation has been settled for a long time, new approaches were and will continue to be achieved for the sake of improving of evaluation and quality control of surfaces in scientific and technological research and in industry. Below we will briefly present the approaches and some applications explored at the Microtopography Laboratory of the Department/Center of Physics of the University of Minho in Portugal. The usefulness of triangulation-based dimensional inspection systems is clearly demonstrated. A recent simple web search revealed over 50 companies that produce and commercialize triangulation-based sensors for a wide range of application resolutions, working distances and dynamic ranges [46].

### 1.2. Optical Triangulation

**Figure 1.**
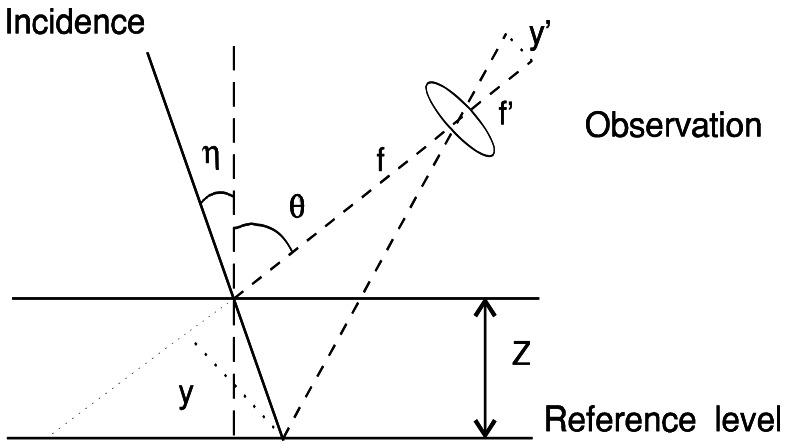
A sketch of the general triangulation geometry.

The general triangulation procedure can be illustrated as in Figure 1. Depth information can be easily obtained. The relation between the measured value (y′) and height (Z) is simply:

(1) $$y\prime =M\frac{Z\sin(\eta +\theta )}{\cos\eta +(\overset{Z}{\;}/\underset{f}{\;})\cot(\eta +\theta )}$$

where M = f′/f is the optical magnification in the observation arm, f and f′ are the focal lengths of the observation optics, η the incidence angle and θ the observation one.

This active triangulation structure illustrated above is geometrically equal to the passive triangulation approach were the incidence arm is replaced by a second observation arm. Triangulation-based dimensional inspection methods were re-invented and new moiré, structured lightning and discreet triangulation systems were developed.

Moiré methods are being used with success since the 70s [47,48] in 3D inspection of objects and topography of surfaces. Moiré topography uses moiré fringes that appear when two gratings of similar frequencies are superimposed. In the two basic approaches, shadow and projection moiré, moiré fringes become contour lines of surfaces in specific geometrical conditions. Unfortunately the identification of the absolute fringe orders due to height differences between consecutive fringes, is still a problem, specially on complex rough surfaces with sharp or discontinuous cuts.

In order to achieve greater accuracy and versatility several approaches were suggested: two-frequency shadow type moiré, moving gratings, phase-shifting holographic moiré, slit beam scanning moiré, color gratings, …, and the use of computer generated gratings has been tried too [49–53].

Without considering interference effects several systems have been developed, and are commercially available, that use fringe projection or in general structured lightning. Typically one light stripe is projected onto the object at a known angle and is viewed by one or two cameras from another(s) angle(s). The stripe is made to scan the sample by mechanical or opto-mechanical means.

More popular with the advent of large pixel number CCD cameras and inexpensive, real time frame grabbers and image processing software, became the projection of not just one stripe at the time but a whole fringe pattern covering virtually all the area to be inspected. Other kinds of patterned lightning are employed like grids or arrays of points. The use of liquid crystal light valves or LCD displays makes the coding process fast and straightforward [3,54,55]. The optical and mechanical complexity of these systems is usually very small, making them quite suitable for industrial applications.

Discreet, active triangulation procedures, where a small diameter or focused well-shaped light beam is projected onto the samples and the areas to be inspected scanned, are also of good use in the industry for their reliability, versatility and friendly use.

Robust sensors using different kinds of illumination, scanning or detection methods are available presenting top resolutions on the submicron level, with high dynamic range and inspection rates of several hundred points per second [3,4,38,56–61].

**Figure 2.**
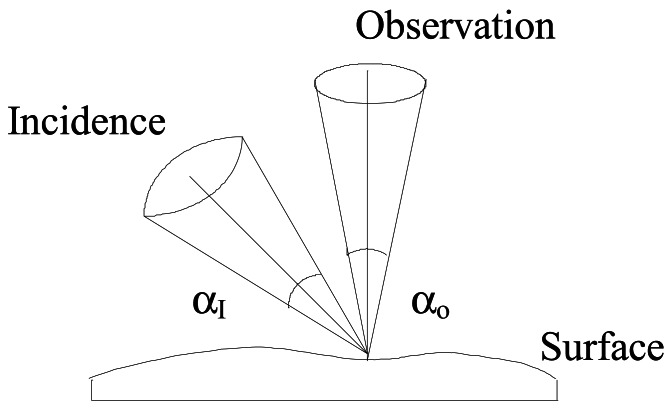
The surface's relief inspection system we implemented is based on the geometry sketched above.

**Figure 3.**
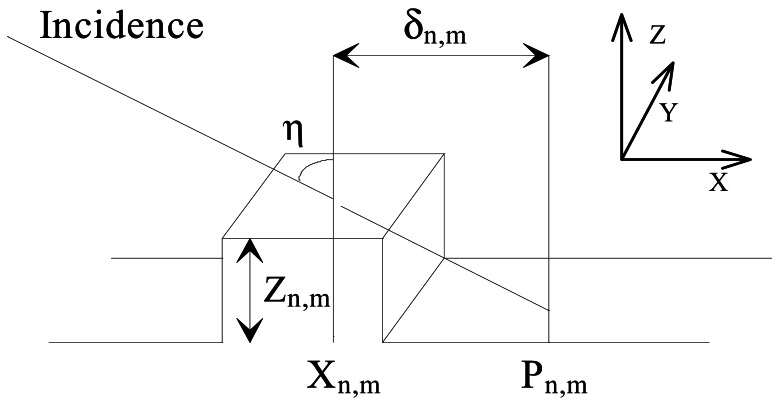
The intersection of an oblique light beam with an opaque surface creates on it a bright spot whose lateral position depends on the surface height.

Typically in sensors based in active discreet triangulation the bright spot that is created on a flat surface on the incidence of an oblique light beam (Figures 2 and 3) moves over the surface when this is given a normal displacement [37]. An observer located above the surface will notice this lateral movement and be able to translate them into the displacement incurred by the surface. Alternatively, if there is a reference level and the sample under observation is placed above it, the lateral movement of the spot can be translated in terms of local thickness.

The sample is placed on a reference surface and scanned by a light beam shinning obliquely, and focused onto a small, diffraction limited, spot on the surface. The scanning is made by moving the sample step by step in equal precisely known increments. At each sampled point, the bright spot's position is imaged on a camera (a linescan camera will be a suitable solution as the spot's position will always lay on the incidence plane) that is interfaced with a microcomputer where the spot's position will be registered. At each scanning step the spot shift regarding the reference position is calculated and the thickness, or height, and the really inspected surface points are computed and identified. The three-dimensional set of co-ordinates (Figure 3) will be:

(2) $$\begin{array}{c} \text{Xn},\text{m}=\text{n}\Delta -\delta \text{n},\text{m};\\ \text{Yn},\text{m}=\text{m}\Phi ;\\ \text{Zn},\text{m}=(\delta \text{n},\text{m}/\text{M})\cot\eta \text{.} \end{array}$$

where η is the incidence angle, Δ the sweep increment in the direction of the plane of incidence (X) and Φ in the perpendicular one, M the magnification of the observation system placed above, perpendicularly, to the surface, and δn,m the spot shift (on the X direction), regarding a reference position Pn,m, at the scan position (Xn,Ym).

The type of the surface under inspection, and its relief characteristic in the three dimensions, condition the overall performance of this method in addition to its particular implementation.

The depth resolution (Zmin) will be conditioned by the speckle effects when laser light is used as happens most commonly, and it will be essentially limited by the Rayleigh limit modified upon the system's particular configuration. Referring to Figures 2 and 3:

(3) $$\text{Zmin}=\lambda /\sin\alpha_{\text{o}})\cot\eta$$

The dynamic range that depends on the system's configuration will vary from around 1:300 to 1:5,000. The lateral resolution depends on the system that will be implemented and will be defined by the maximum scanning resolution and the spot's size and configuration.

In order to overcome triangulation's inherent problem of shadowing and mutual reflection, especially when we use large incident angles that give the best resolution, we scan the sample consecutively with two opposite angles (η and −η). The results are then matched and the final set of three dimensional coordinates is obtained [37].

## 2. Triangulation Based Microtopographic Inspection at the University of Minho

**Figure 4.**
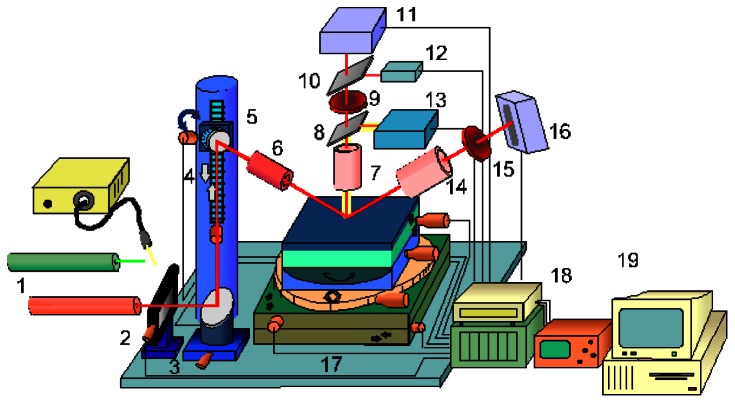
Sketch of the MICROTOP.06.MFC system: 1—Interchangeable light sources; 2—Vibration isolation stand; 3—Neutral density filter; 4—Beam steering system; 5—Incidence angle control motorised system; 6—Incidence optics; 7—Normal observation optics; 8 and 9—Beam splitters; 10—Interference filter; 11—Normal photosensitive detection system; 12—Photodetector; 13—Video camera and illuminator; 14—Specular observation optics; 15—Interference filter; 16—Specular photosensitive detection system; 17—Sample support and motorised positioning system; 18—Data acquisition and control system; 19—Microcomputer.

For several years a range of optical profilometers and microtopographers, the MICROTOP' family, were developed at the Microtopography Laboratory of the Physics Department of the Universidade do Minho, Portugal aiming at different applications, and improved and adapted according to particular inspection needs [38,62–79]. Several systems are available: the MICROTOP.03.MFC is intended for general use in the inspection of rough surfaces (both 2D and 3D); the MICROTOP.PL1.MFC system is a simplified version specially designed to be used in the inspection of polymer pieces and materials [70,73]; a simple hand held version [38]; and the version MICROTOP.06.MFC (Figure 4) that incorporates an angle resolved scattering structure with height resolution measuring capabilities down to the nanometer range [77].

**Figure 5.**
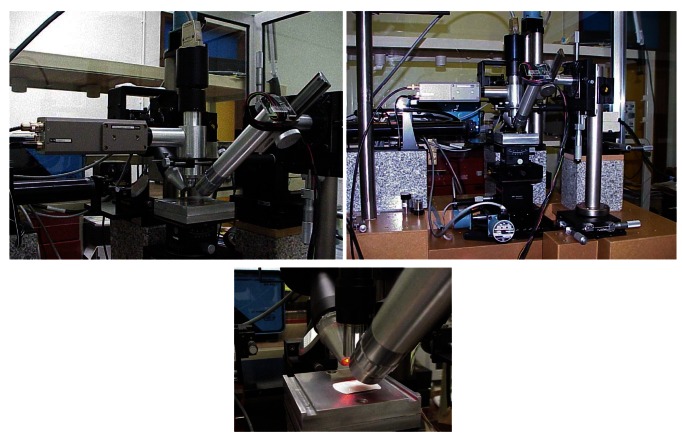
Views of the MICROTOP.06.MFC setup.

The MICROTOP.06.MFC is an improved version of the MICROTOP.03.MFC system incorporating a number of innovative features. Increased versatility, reliability, with larger measuring range, better accuracy and resolution that now can be driven down to the nanometer range, were achieved in the MICROTOP.06.MFC. The MICROTOP.03.MFC is based on a method involving optical active triangulation with oblique incidence and normal observation, and mechanical sample's scanning (Figures 2 and 3). However it can be easily reconfigured by changing the observation and incidence arms and reducing the triangulation arm allowing the inspection rougher surfaces or objects with higher height ranges [76]. In the MICROTOP.06.MFC version another triangulation arm is incorporated on the sensor's head allowing specular triangulation with resolutions down to the nanometer range [75]. Furthermore when using a linescan scan camera with 2,048 elements, pitch 13 μm, the roughness of smoother samples can be measured by an angular resolved scattering approach [77,78]. If on the specular observation arm a differential photodiode is employed resolutions of a few nanometers are achieved on the inspection of smooth surfaces. A CCD camera with a coaxial illuminator allows 2D images to be acquired and processed [80]. In the MIROTOPO.06.MFC the method was extended by incorporating angle resolved scattering methods. The setup and the inspection process are briefly described next referring to Figure 4. In Figure 5 a few pictures of the actual system are presented.

The surface to be inspected is scanned by one oblique light beam. Different light sources are available (two HeNe lasers at 632.8 and 534 nm, and, one Xe white light source) and can be easily interchanged. The incident light is collimated and focused. A small, diffraction limited, bright spot is thus projected onto the sample. The bright spot is imaged both perpendicularly and specularly onto electronic photosensitive detection systems in order to assess its lateral position. As sensors several options are available to be chosen according to the required application: one 2,048 pixel Fairchild CCD linear array on the specular arm and a Reticon line scan camera; one PSD; and a differential detector. The area of the surface to be inspected is scanned point by point by the “sensor's tip” (the light beam focused onto the surface). The highest system's robustness was sought. Also a high lateral positioning resolution and accuracy should be achieved. Thus both the incidence arm and observation arms of the sensor are kept fixed. In order to perform the sample's scanning it will be moved by means of a precision XY displacement table driven by precision step motors. Piezo-driven motors allow positioning with nanometer resolution in a 1.5 mm range. At each scanning point, on a rectangular array separated by distances down to 1.25 μm, the lateral spot's position in both sensors is obtained and registered. The spot's shift on both detectors' planes, between consecutive scan positions is directly related with the height differences between those surface' inspected points. In the “specular” arm of the system the detector can be positioned (just introducing an adapter) tilted relative to the observation optics in order to increase the depth range of the sensor (Schleimpflug' condition). Employing the linear arrays both arms are on a confocal arrangement allowing the best resolution.

The incidence set-up comprises apart from the light source a neutral density variable filter, a motorised beam steering system, a spatial filter and focusing optics. The change on the incidence angle is made synchronized with the change of the observation angle on the specular arm. A vertical movement precision stage endowed with computer controlled motion provided by a reliable accurate DC encoder with high positioning repeatability and resolution is used for refocusing the observation optical system but especially for calibration of both arms of the sensor. In order to resolve shaded areas and mutual reflections, a high precision rotational stage is used allowing easy change to opposite light incidence. Often the faces of the surface to be analyzed are not parallel or simply the surface to be inspected does not lie horizontally. In order to maintain the best height resolution a tilt table was incorporated to the samples' positioning system. Furthermore it may allow the inspection of 3D objects or surfaces with pronounced holes of it, for instance.

The observation optical systems are formed by microscope objectives chosen according to the characteristics of the surface's relief. In both sensor' arms the objectives can be independently focused. They will be used to image the light spot onto the opto-electronic photosensitive detection systems. Both the “normal” and the “specular” sensors' arms are attached to a XYZ precision displacement table for finer adjustments. A 2D CCD camera was attached to the system allowing the capture of bi-dimensional color images of the scanned area for matching and as an improved visualization aid. Projection of the actual 2D image onto the 3D map is being studied at the moment. In order to cope with different requirement different photosensitive systems are available and all are interchangeable. A personal microcomputer acquires the data and takes control of the whole inspection process and result's presentation. At the end of the inspection process we may have just one but typically will have two sets of data one for each sensor's arm. Data processing is independently performed and two sets of parameters and functions are obtained by triangulation and scattering analysis. The correlation of the sets of data is investigated. Comparison and matching is performed in order to obtain just one the best set of reliable and accurate data.

### A Few Application Examples

**Figure 6.**
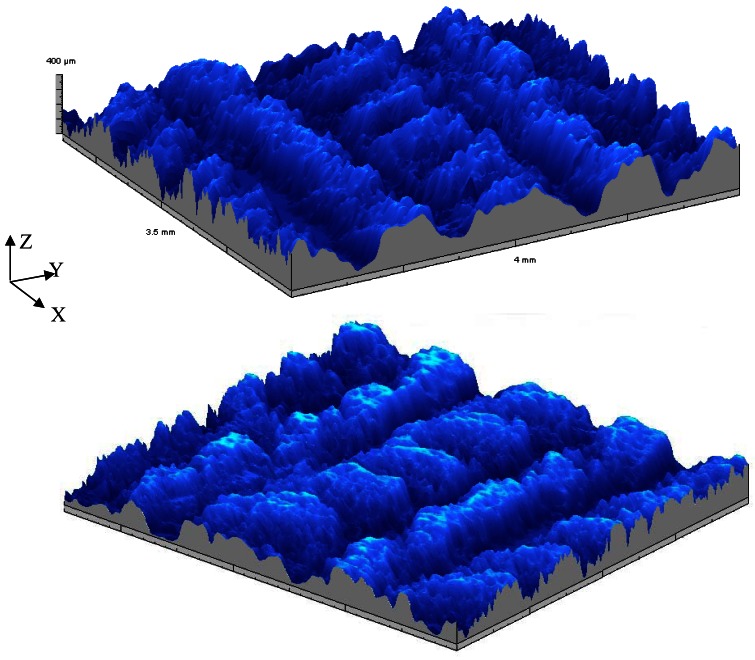
Relief map obtained with the MICROTOP.03.MFC system of a 3.5 × 4 mm^2^ sample of linen fabric. When viewed from different directions a better understanding of the surface topography can be achieved.

Over last two decades a large number of different applications of the microtopographer developed at the Microtopography Laboratory of the Department of Physics of the University of Minho were successfully performed [60–80]. In the first application we were requested to measure the roughness of fabrics evolving later to the tri-dimensional inspection of different kind of fabrics [62]. In Figure 6 the relief map, obtained with the MICROTOP.03.MFC, of a 3.5 × 4 mm^2^ sample of linen fabric is shown. The anisotropy due to the crossing of the linen threads is clearly evident. In order to produce a X-ray detector array, cavities to old scintillation crystals were open by laser ablation (KrF excimer laser) on aluminum sheets using different values of fluency, frequency and pulse length.

**Figure 7.**
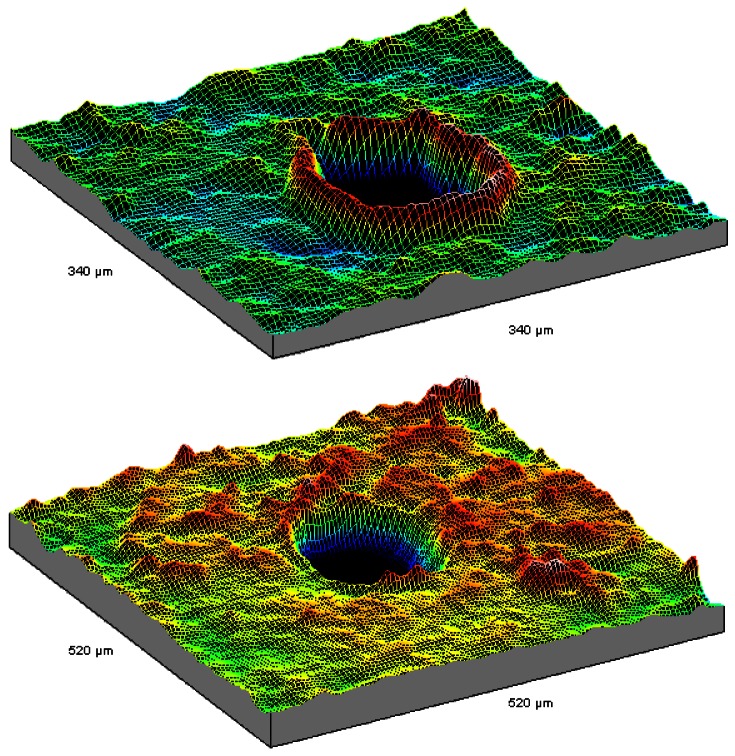
3D maps of cavities open by laser ablation on 0.5 mm tick aluminum sheets.

Two relief maps are shown in Figure 7 of ∼100 μm diameter holes obtained with 5250 10 mJ laser shots at 10 Hz and at 4 Hz. Lower frequencies seem to produce better results [67].

**Figure 8.**
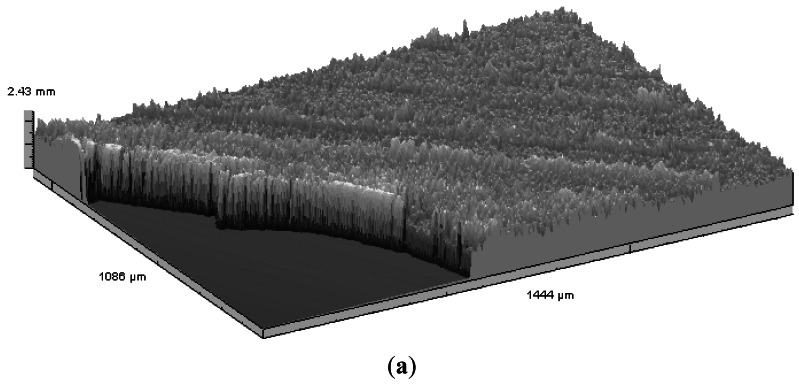

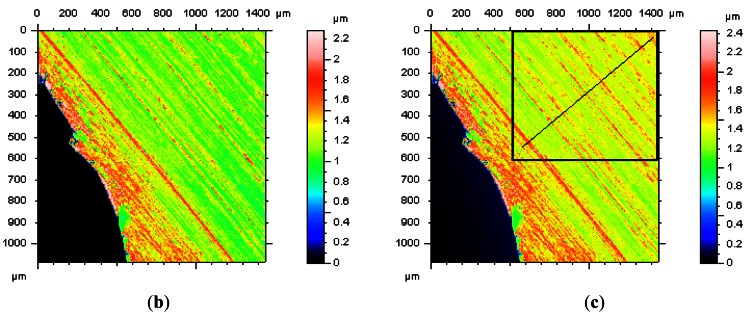
Microtopographic inspection of a 2 micron thick mirror like aluminium coating presenting a lateral fracture. The measures were performed with the inspection system configured to a resolution of 7 nm. On the right bottom image is marked an area away from the fracture zone were roughness of the coating' surface was measured (Figure 9).

**Figure 9.**
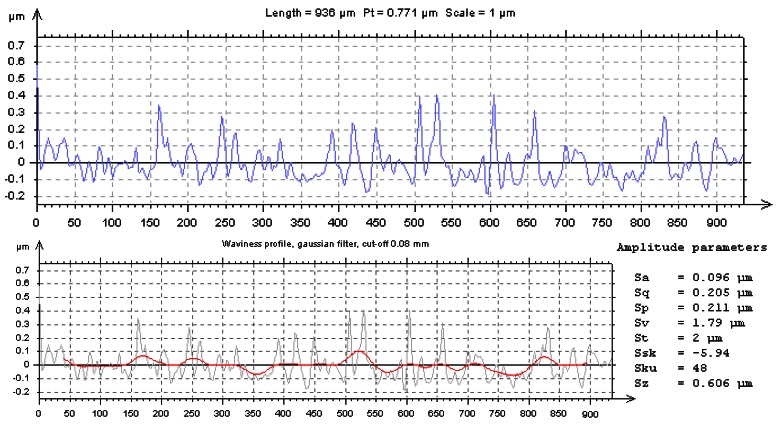
Profile on the oblique line sketched on the right map of Figure 8(c) is shown here presenting also, below, the waviness profile obtained applying a double a gaussian filter and a 0.08 mm cut-off (red line). Statistical amplitude parameter obtained from the inspection of an area of the original relief map away from the fracture region (the rectangle on the right map of Figure 8).

The Department of Physics of the University of Minho has been very active from the late 80s in the production and characterization of coatings and thin films of different types and purposes. The rugometric and microtopographic inspection of different samples were performed [64–66,68,69]. In Figures 8 and 9 are presented a few results of the microtopographic inspection and roughness characterization of a 2 micron thick mirror-like aluminum coating presenting a lateral fracture. The measurements were performed with the inspection system configured to allow a height resolution of 7 nm. The 3D rms roughness, Sq, obtained from the inspection of a region of the film away from the fracture region (Figure 8(c)) is in fair agreement with the value obtained by averaged pseudo ARS (0.213 ± 0.011 μm) [81].

From the coatings surfaces' relief maps obtained with our laser microtopographer we are also able to calculate the coatings' residual stress in that particular coating/substrate system [77].

**Figure 10.**
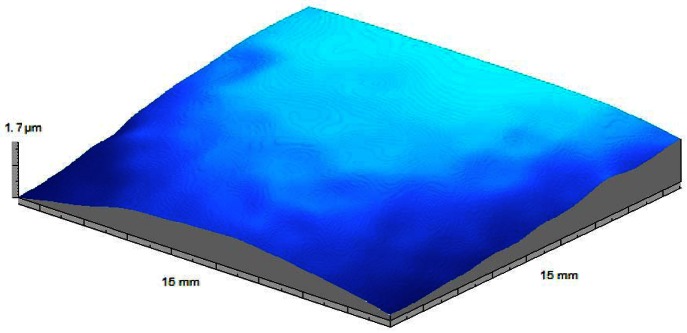
Relief map of a WO_3_ film used in electro-chromic applications [77].

**Figure 11.**
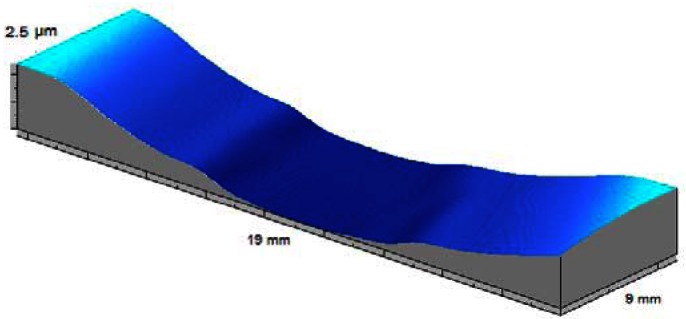
Microtopographic map of a tungsten film. Different curvatures in central and outer regions of the film are noticeable possibly due to partial non adherence of the film to the substrate.

In Figure 10 we present the results of the stress evaluation of a 123 nm thick β tungsten thin film PVD deposited, on a 20 mm per 20 mm square glass blade 0.15 mm thick, at a 4 × 10^−2^ mbar pressure during 6 minutes in a sputtering chamber. The substrate-target distance was set to 60 mm and the sputtering current of 0.23 A. The thin film/substrate system presents a slight curvature and a surface with an average roughness (Ra) of 0.34 μm. The calculated transversal compressive stress ranges between 461 and 447 MPa (454.38 ± 6.84 MPa) across the central region of the film. This roughly 1.5% variation is not significant and the stress distribution is essentially isotropic. The three-dimensional topographic inspection of the sample allows easier detection of any anisotropy in the residual stress distribution. Furthermore we can also distinguish zones in the coating that may eventually present different residual stress for instance off the central region of the film. This is what happens in the film represented in Figure 11. The tungsten film produced a major curvature in the glass substrate. High stress caused a partial non adherence of the film to the substrate. Despite the fact the detachment is evident the residual stress calculated in the central zone (still adhered to the substrate) is only 9% higher than at outer regions (510.7 and 459.8 MPa respectively).

**Figure 12.**
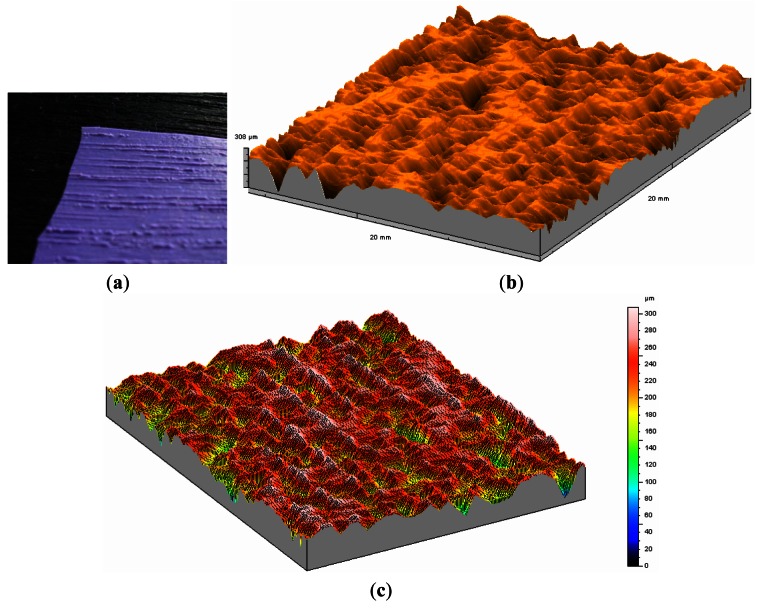
A blackwood plate replica (**a**) and results of its microtopographic inspection (graphs (**b**) and (**c**)).

**Figure 13.**
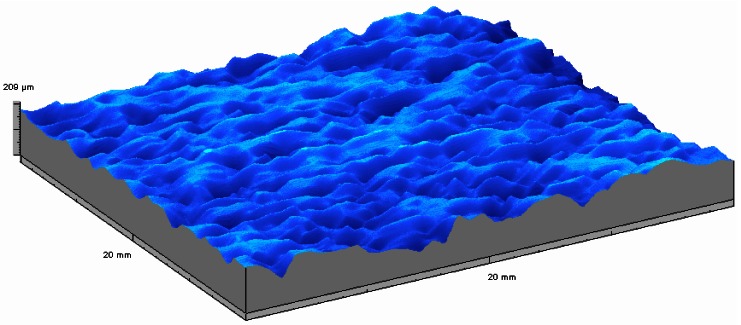
Relief map of the varnished face of blackwood plate presented at Figure 12.

In order to illustrate the potential of the system on the rugometric and 3D inspection of wood art and heritage samples, a reduced set of results are presented of the inspection art wood objects furniture or materials for construction. The large variety of types of wood in terms of relief, aspect or surface treatment, including varnishing, painting or gold coating for instance, demands different metrological strategies. In order to be able to measure objects of large dimensions or that cannot be moved portable systems can be used. However when higher lateral and height resolution is sought one can instead obtain high quality surface replicas [71] that can then be carefully inspected with the MICROTOP.03.MFC. Figure 12 a photo shows a blackwood plate and the silicone replica obtained (Figure 12(a)) as well as two relief maps of that surface (different views). One of the faces of the blackwood plate is varnished. In Figure 13 a relief map of the varnished surface is present for comparison. The varnishing process produces a significant decrease on surface's roughness filling up at a significant level the pronounced valleys on the raw surface (Figure 12). In Table 1 we quantify the differences in the most commonly used statistical parameters (3D) the system calculates.

**Figure 14.**
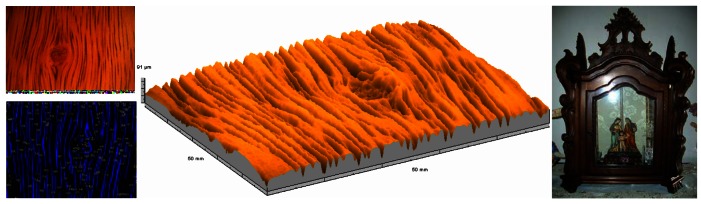
Bi- and tri-dimensional inspection of a chestnut tree wood flat panel on a late XIX century Portuguese “*mostruário*”.

Chestnut tree wood was widely used in Portugal both in the production of quality furniture, statues and other art pieces. A larger variety of art pieces are available on museums all over the country. Often the microtopographic inspection should be complemented by its 2D aspect characterization. In Figure 14 a 2D picture (top left), results of a blob analysis (bottom left) and relief map of a flat panel on a late XIX century Portuguese “*mostruário*” (photo on the right) used to preserve and show in churches, museums and upper class homes religious statues or icons.

**Figure 15.**
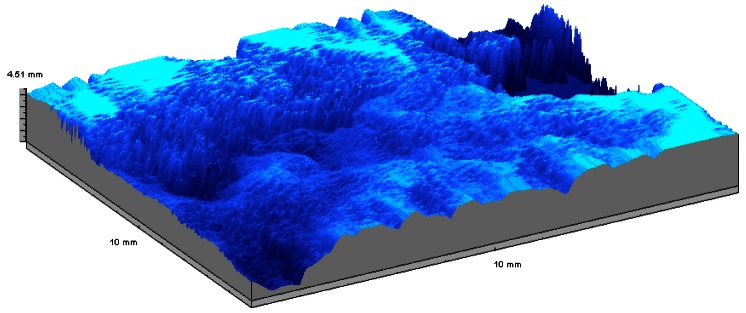
Relief of the surface of fracture of a granite test block obtained in an area of 1 cm^2^.

3D objects and much rougher surfaces were also inspected. The surfaces of fracture of granite blocks subjected to tensile stress present topographic structures with Rt roughness up to several millimetres [76]. These relatively high values demanded a reconfiguration of the MICROTOP.03.MFC by exchanging the observation and incidence arms of the system and reducing the triangulation angle. A typical fracture surface relief map is presented in Figure 15.

**Figure 16.**
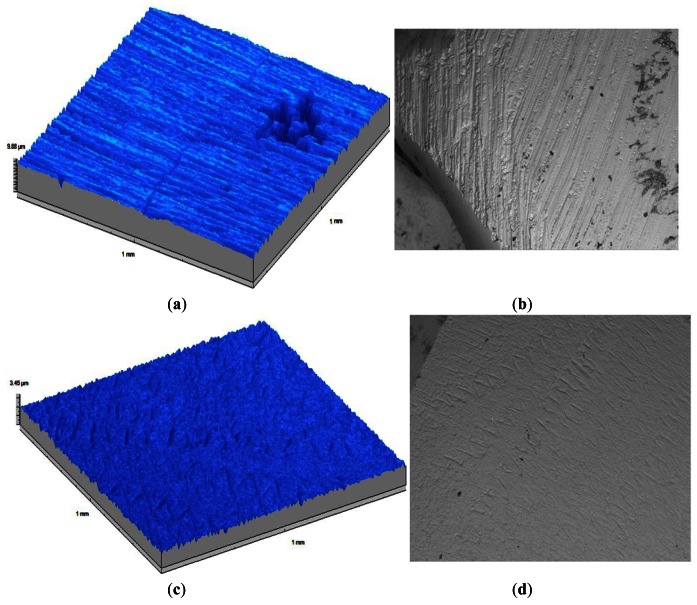
Relief maps (1 mm^2^) and SEM images (on the right) of teeth surface samples stripped with the tungsten carbide bur (**a,b**) and the Ortho-Strips^®^ 90-40-25-15 (**c,d**) orthodontic techniques. Although the average roughness is similar the total roughness, Rt, is lower with the later technique therefore giving the indication that it is the most adequate technique.

We have also proven that the rugometric and microtopographic inspection by non-invasive optical active triangulation-based microtopography provides valuable insights into the choice of the most adequate stripping technique on orthodontic teeth interproximal reduction [79], a technique widely used in dentistry. In order to evaluate the quality of dental enamel stripping the treated tooth surface was topographically inspected in order to gather meaningful statistical surface characterization parameters like average roughness, Ra. The particular tri-dimensional shape of human tooth is somewhat complex and furthermore the area that needs to be subjected to stripping is frequently rather small and usually non flat making it particularly difficult to proceed with the rugometric and microtopographic inspection [77]. In Figure 16 relief maps (1 mm^2^) of samples treated with different standard methods used in orthodonthics are shown, as well as SEM images of the same teeth surfaces.

**Figure 17.**
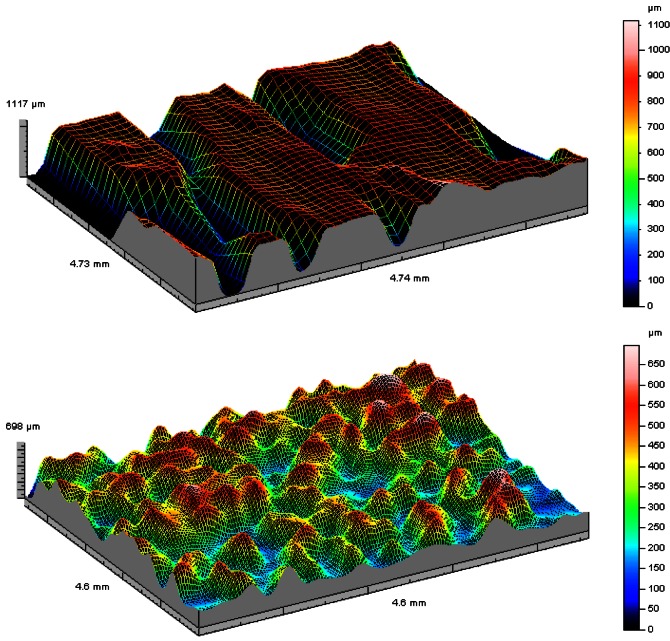
Three-dimensional maps of latex replicas of a patient with melanoma: above on a healthy area, and below in the injured area. In the injured area the skin marks in the healthy skin disappears in a much rougher surface.

Another successful application of our inspection systems in life sciences was in the study of the relation between the surface roughness of skin marks and skin cancer type and malignity allowing the development of a new tool for early skin cancer diagnosis. In Figure 17 we show, as an example, two 3D relief maps of skin latex replicas of a patient with melanoma. The map on the top refers to the replica of a healthy area, and the one below to the topography of an injured area. In the injured area the deep pronounced lines in the healthy skin disappears in a much rougher surface.

From the microtopographical inspection of cancerous skin, characteristic information could be obtained that enables the differentiation among the types of lesion studied. For the melanoma it was observed that on average these tumors show an average roughness increased of 67% compared to the average roughness of the healthy skin. These measures allow them to be distinguished clearly from other tumors as in the case of the basal cell carcinoma (49%) and benign lesions like the epidermoid cyst (37%) and the seborrhea keratosis (4%). We also observed that when the tumor is bigger and rougher it is more malignant.

## 3. Conclusions

Optical triangulation has for a long time proved to be an invaluable tool in microtopographic evaluation of surfaces and structures in scientific research and in the industrial environment. In different approaches and implementations it is an invaluable tool in quality control and dimensional metrology. New light sources, electronic sensors and optical and electro-optical elements will allow further developments. The MICROTOP family of active triangulation-based surface inspection systems developed at the Microtopography Laboratory of the University of Minho has been tested in a number of different applications and metrological tasks with its results being confirmed by different commercially available systems. These tests have extensively demonstrated its accuracy, versatility robustness and reliability.

## Figures and Tables

**Table 1.** Comparison of roughness parameters of a black wood sample after and before varnishing.

**Without Varnishing**	**Varnished**	**Difference (%)**

Sa = 51.96 μm	Sa = 47.47 μm	8.6
Sq = 69.74 μm	Sq = 58.97 μm	15.4
Sz = 303.89 μm	Sz = 208.79 μm	31.3
Sm = 403.13 μm	Sm = 2,462.24 μm	
Ssk = −1.59	Ssk = −1.08	
Sku = 4.99	Sku = 2.98	
